# Postoperative clinical outcomes for kinematically, restricted kinematically, or mechanically aligned total knee arthroplasty: a systematic review and network meta-analysis of randomized controlled trials

**DOI:** 10.1186/s12891-023-06448-0

**Published:** 2023-04-24

**Authors:** Takanori Miura, Tsuneari Takahashi, Jun Watanabe, Yuki Kataoka, Ryusuke Ae, Hidetomo Saito, Katsushi Takeshita, Naohisa Miyakoshi

**Affiliations:** 1 Department of Orthopedic Surgery, Tazawako Hospital, 17-1 Ukiyozaka Obonai, Tazawako, Senboku, Akita 014-1201 Japan; 2 Department of Orthopedic Surgery, Ishibashi General Hospital, 1-15-4 Shimokoyama, Shimotsuke, Tochigi 329-0596 Japan; 3 Department of Surgery, Division of Gastroenterological, General and Transplant Surgery, Jichi Medical University, 3311-1 Yakushiji, Shimotsuke City, Tochigi 329-0498 Japan; 4 Center for Community Medicine, Jichi Medical University, 3311-1 Yakushiji, Shimotsuke City, Tochigi 329-0498 Japan; 5 Scientific Research WorkS Peer Support Group (SRWS-PSG), Osaka, Japan; 6 Department of Internal Medicine, Kyoto Min-Iren Asukai Hospital, Tanaka Asukai-Cho 89, Sakyo-Ku, Kyoto, 606-8226 Japan; 7 Section of Clinical Epidemiology, Department of Community Medicine, Kyoto University Graduate School of Medicine, Yoshida Konoe-Cho, Sakyo-Ku, Kyoto, 606-8501 Japan; 8 Department of Healthcare Epidemiology, Kyoto University Graduate School of Medicine / Public Health, Yoshida Konoe-Cho, Sakyo-Ku, Kyoto, 606-8501 Japan; 9 Division of Public Health, Center for Community Medicine, Jichi Medical University, 3311-1 Yakushiji, Shimotsuke City, Tochigi 329-0498 Japan; 10 Department of Orthopedic Surgery, Akita University Graduate School of Medicine, 1-1-1 Hondo, Akita, 010-8543 Japan; 11 Department of Orthopaedic Surgery, School of Medicine, Jichi Medical University, 3311-1 Yakushiji, Shimotsuke, 329-0498 Japan

**Keywords:** Total knee replacement, Total knee arthroplasty, Mechanical alignment, Kinematic alignment, Restricted kinematic alignment, Systematic review, Network meta-analysis

## Abstract

**Background:**

Mechanically aligned total knee arthroplasty (MATKA) is a well-established procedure. Kinematically aligned TKA (KATKA) has been proposed to restore and preserve pre-arthritic knee anatomy. However, normal knee anatomy varies widely, and there have been concerns regarding restoring unusual anatomy. Accordingly, restricted KATKA (rKATKA) was introduced to reproduce constitutional knee anatomy within a safe range. This network meta-analysis (NMA) aimed to evaluate the clinical and radiological outcomes of the surgeries.

**Methods:**

We performed a database search on August 20, 2022, which included randomized controlled trials (RCTs) comparing any two of the three surgical TKA techniques for knee osteoarthritis. We conducted a random-effects NMA within the frequentist framework and evaluated confidence in each outcome using the Confidence in Network Meta-Analysis tool.

**Results:**

Ten RCTs with 1,008 knees and a median follow-up period of 1.5 years were included. The three methods might result in little to no difference in range of motion (ROM) between methods. In patient-reported outcome measures (PROMs), the KATKA might result in a slight improvement compared with the MATKA (standardized mean difference, 0.47; 95% confidence interval [CI], 0.16–0.78; very low confidence). There was little to no difference in revision risk between MATKA and KATKA. KATKA and rKATKA showed a slight valgus femoral component (mean difference [MD], -1.35; 95% CI, -1.95–[-0.75]; very low confidence; and MD, -1.72; 95% CI, -2.63–[-0.81]; very low confidence, respectively) and a slight varus tibial component (MD, 2.23; 95% CI, 1.22–3.24; very low confidence; and MD, 1.25; 95% CI, 0.01–2.49; very low confidence, respectively) compared with MATKA. Tibial component inclination and hip–knee–ankle angle might result in little to no difference between the three procedures.

**Conclusions:**

KATKA and rKATKA showed similar ROM and PROMs and a slight variation in the coronal component alignment compared with MATKA. KATKA and rKATKA are acceptable methods in short- to mid-term follow-up periods. However, long-term clinical results in patients with severe varus deformity are still lacking. Surgeons should choose surgical procedures carefully. Further trials are warranted to evaluate the efficacy, safety, and subsequent revision risk.

**Supplementary Information:**

The online version contains supplementary material available at 10.1186/s12891-023-06448-0.

## Background

For end-stage knee osteoarthritis (KOA) patients, total knee arthroplasty (TKA) is an established surgical procedure that alleviates pain and improves knee function. Its use is expected to increase owing to its excellent clinical results [1].

Mechanically aligned TKA (MATKA) is the gold standard, and the satisfaction rate associated with this procedure is generally high [2]. However, some patients express dissatisfaction after MATKA, the reasons for which are multifactorial and remain poorly understood and unresolved [3, 4]. Recently, restoring and preserving pre-arthritic knee anatomy during TKA has gained increasing attention, and kinematically aligned total knee arthroplasty (KATKA) has been proposed [5].

As a result of restoring the knee to the patient’s native alignment, good soft tissue balance and similarity to the native kinematics are expected to be restored [6].

Compared with conventional methods, KATKA may achieve improved physiological joint line obliquity and reduce the need for ligament release to produce a balanced joint [5, 7, 8]. Despite the advantages of KATKA, which may lead to better clinical outcomes [5], component loosening, especially in the tibia owing to varus placement, is a potential risk factor for this procedure [9–11]. The degree of outlier range of alignment, which may induce shorter-term implant survivorship using current TKA methods, is unknown. Postoperative alignment with a deviation of > 3° has no effects on long-term survivorship [12]. Howell et al. reported an excellent implant survival rate of 97.5% at the 10-year follow-up in a cohort of 222 knees after KATKA [13]. In contrast, small increases in the tibial component varus, compared with native alignment, were associated with early aseptic revision in patients undergoing KATKA. Increasing the tibial varus by ≥ 2.2° is associated with an increased likelihood of revision [14]. Therefore, KATKA is a controversial procedure in some patients [15].

Accordingly, a modified KATKA technique, the restricted KATKA (rKATKA), involves modifications using bony cuts within a “safe range,” as defined by the following criteria: independent tibial and femoral cuts must be within ± 5° of the mechanical axis of the respective bone, and the resulting overall hip–knee–ankle angle (HKA) must fall within ± 3° of neutral alignment [16]. A randomized controlled trial (RCT) revealed that rKATKA improved knee balance, as indicated by a reduced number of knee-balancing procedures, compared with MATKA [17]. Several systematic reviews comparing the clinical outcomes of MATKA and KATKA and those of MATKA and rKATKA revealed similar results without an increased risk of implant failure [15, 18–21]. Furthermore, the difference between KATKA and rKATKA is clinically crucial; however, no RCTs or systematic reviews have compared these methods. A network meta-analysis (NMA) expands upon the concept of the traditional meta-analysis by producing pairwise comparisons and demonstrating the relative treatment effects across a range of interventions through direct and indirect comparisons [22]. Thus, this NMA aimed to compare the clinical outcomes of MATKA, KATKA, and rKATKA.

## Methods

### Protocol and registration

The study protocol was developed in accordance with the Preferred Reporting Items for Systematic Review and Meta-Analysis 2020 (PRISMA-2020) and PRISMA for Network Meta-Analyses (PRISMA-NMA) [23, 24]. We have registered our protocol in the Open Science Forum (https://osf.io/2q4pr/). Additional file 1 shows the PRISMA checklist.

### Inclusion criteria of the articles for the review

#### Type of studies

We included individual RCTs comparing any two of the surgical techniques of TKA for KOA with the aim of comparing the clinical outcomes of MATKA, KATKA, and rKATKA.To ensure a comprehensive evaluation of the evidence, it was necessary to include both published and unpublished studies. To minimize any potential for publication bias, we followed the guidelines set out in the Cochrane handbook, we included all published studies, unpublished articles, conference abstracts, and letters without applying language or country restrictions. Additionally we did not exclude studies based on observation period or publication year.

### Study participants

We included adult patients aged > 18 years who underwent primary TKA for primary or secondary KOA. The surgical techniques were defined as follows: MATKA, TKA performed to achieve neutral mechanical limb alignment (0° HKA), an anatomic axis of the knee (femoral–tibial angle) within -2.5° ± -7.4° valgus, and a varus–valgus angle of the tibial component perpendicular to the tibial mechanical axis [12]; KATKA, TKA performed to achieve natural alignment by removing a cartilage and bone thickness equivalent to the implant thickness and positioning the femoral and tibial components to the angles and levels of the distal and posterior femoral joint lines and tibial joint line [5]; and rKATKA, TKA performed using bony cuts within a “safe range” as defined by certain criteria, including independent tibial and femoral cuts within ± 5° of the mechanical axis of the respective bone and the overall resulting HKA falling within ± 3° of neutral alignment [16].

### Outcomes of interest

The primary outcomes were postoperative range of motion (ROM), patient-reported outcome measures (PROMs), and revision surgeries. The secondary outcomes were component and lower limb alignment and all adverse events. We selected the Western Ontario and McMaster Universities Osteoarthritis Index (WOMAC, 0 [best] to 96 [worst]) score [25], Oxford knee score (OKS, 0 [worst] to 48 [best]) [26], and Knee Injury and Osteoarthritis Outcome Score (KOOS, 0 [worst] to 100 [best]) [27]. In the case of multiple PROMs used in one study, we used WOMAC, a widely used and validated patient-administered questionnaire that assesses pain, stiffness, and function for integration [25, 28]. We evaluated postoperative radiological outcomes as follows: (1) femoral component alignment (relative to mechanical axis and + means varus), (2) tibial component alignment (relative to mechanical axis and + means varus); (3) tibial component inclination; (4) the HKA, the angulation between the mechanical axes of the femur and tibia by radiograph and—means varus [29]. All adverse events were followed according to the definitions of the original authors.

### Search strategy

We searched the Cochrane Central Register of Controlled Trials (CENTRAL), MEDLINE via PubMed, EMBASE via Dialog, World Health Organization International Clinical Trials Registry Platform, and ClinicalTrials.gov on August 20, 2022, using specific keywords (Additional file 2). We also screened the reference lists and the cited articles of all included studies, including international guidelines for TKA, using a citation chaser [30–32].

### Study selection and data extraction

Two independent reviewers (T.M. and T.T.) assessed the identified publications for eligibility. We extracted data from the included studies using a standardized data collection form. Preoperative limb alignment, insert selection (cruciate retaining [CR], posterior stabilized [PS], or others), and surgical approach (medial parapatellar [MPP] or other approaches) were also investigated as effect modifiers. We contacted the original authors for missing relevant unpublished or additional data. Any disagreements between the two reviewers were resolved through discussion; if no consensus was reached, a third reviewer arbitrated (J.W. or Y.K.).

### Geometry of the network

Network geometries are depicted. Circles represent the surgical procedure as a node in the network, lines represent direct comparisons using studies, and the width of lines represents the number of studies included in each comparison, also represented by numbers.

### Risk of bias assessment

Two reviewers (T.M. and T.T.) independently evaluated the risk of bias using the Risk of Bias (ROB) 2 tool [33, 34]. We intended to evaluate the effect assignment to the interventions with an “intention-to-treat” effect. Disagreements between the two reviewers were discussed, and if no consensus was reached, a third reviewer (J.W. or Y.K.) arbitrated.

### Data synthesis and statistical analysis

We pooled the odds ratios (OR) and 95% confidence intervals (CI) for revision surgery. We also pooled the mean differences (MD) and 95% CI or standardized mean differences (SMD) for continuous variables using pairwise comparisons and NMA. We summarized the adverse events; however, we did not perform a meta-analysis. An NMA of the outcomes of the three surgical techniques utilizing the frequentist model was performed using EZR version 1.55 (Saitama Medical Center, Jichi Medical University, Saitama, Japan), a graphical user interface for R (The R Foundation for Statistical Computing, Vienna, Austria) [35]. We used group-level data; binomial and normal likelihoods were used for dichotomous and continuous outcomes, respectively. We synthesized the study effect sizes using a random-effects NMA model. We also examined the rank of treatments for each outcome using the P-score, which proposes a frequentist analog to the surface under the cumulative ranking curve. The P-score would be 100% when a specific treatment is the best and 0% when it is the worst [36].

### Assessing transitivity

The assumption of transitivity underlying the NMA was evaluated by comparing the distribution of clinical and methodological variables that could act as effect modifiers across treatment comparisons, such as preoperative limb alignment, surgical approach, and implant selection. We assessed the assumption of transitivity for the entire dataset in the final NMA.

### Assessment of the confidence for each outcome

Two reviewers (T.M. and T.T.) evaluated the confidence in each outcome using the Confidence in Network Meta-Analysis (CINeMA) tool [37, 38]. Any disagreements were resolved by discussion, and if no consensus was reached, a third reviewer arbitrated (J.W. or Y.K.). The CINeMA framework includes within-study bias, across-study bias, indirectness, imprecision, heterogeneity, and incoherence. For within-study bias and indirectness, CINeMA calculates the contribution of each study in each network estimate. It combines these contributions with the study-specific evaluations (very low, low, moderate, and high) to rate the relative effect for each comparison in the network.

### Additional analyses

We conducted a subgroup analysis of the PROMs of the following primary outcomes: (1) implant selection (limited to using CR insert studies) and (2) surgical approach (limited to MPP approach studies). Furthermore, we conducted a sensitivity analysis including only studies with a follow-up duration of more than one year.

### Difference between protocol and review

We used EZR in the statistical analysis, and P-scores were used to examine treatment rank. We did not conduct a sensitivity analysis to exclude studies using imputed statistics because none were applicable.

## Results

### Search results and characteristics of included studies

**Fig. 1:**
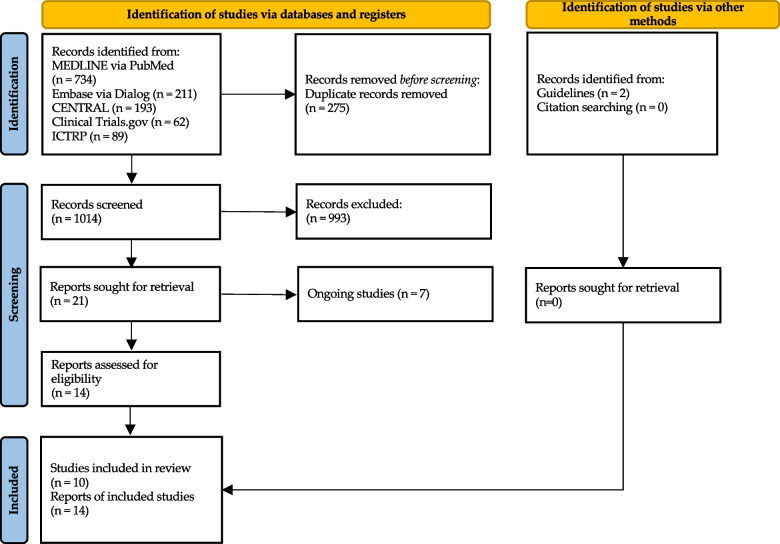
PRISMA flow diagram of the literature search results. CENTRAL, Cochrane Central Register of Controlled Trials; and ICTRP, World Health Organization International Clinical Trials Registry Platform

**Fig. 2:**
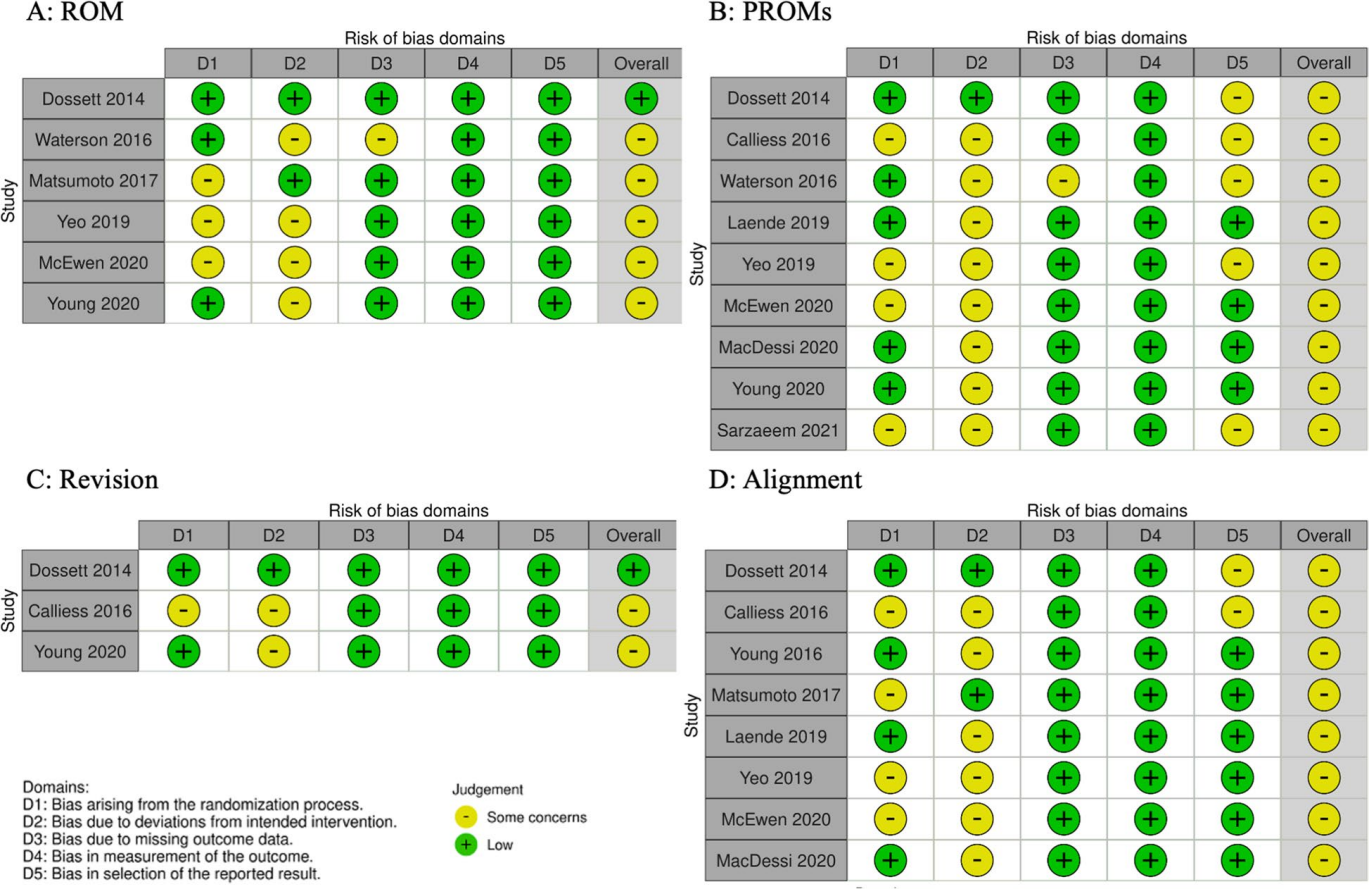
Risk of bias assessment of the included RCTs. Risk of bias graphs for ROM (**A**), PROMs (**B**), revision (**C**), and radiological alignment (**D**). RCTs, randomized controlled trials; ROM, range of motion; PROMs, patient-reported outcome measures

We identified 1,291 records. After conducting a full-text review, we included 10 individual RCTs involving 1,008 knees. We then incorporated these RCTs into the NMA (Fig. 1). Additional file 3 shows the list of studies excluded from this review and the reasons for exclusion. The characteristics and risk of bias assessments of the included studies are shown in Table 1 and Fig. 2, respectively. The median follow-up period was 1.5 years. Seven studies compared KATKA and MATKA [39–48], and three compared rKATKA and MATKA [7, 17, 49, 50]. No study compared KATKA and rKATKA. Only one study showed a varus angle of > 10° in preoperative coronal alignment [7]. For the ROB assessment, most of the studies fell into the “some concerns” category.

**Table 1:** Characteristics of included studies

Authors (Ref. no.)	Year	Country	Compa- rison	Knees		Age (y) (SD)		Female (%)		F/U (y)	Coronal alignment (SD)		Surgical approach	Insert design
				Interve- ntion	control	Interve- ntion	control	Interve- ntion	control		Interve- ntion	control		
Dossett [39, 40]	2014	United States	KA/MA	44	44	66 .0 (7.7)	66.0 (8.6)	4.7	11.7	2	1.5 (5.2)	1.8 (6.5)	NR	CR
Calliess [41]	2016	Germany	KA/MA	100	100	67.0 (8.0)	70.0 (8.0)	61.0	57.0	1	2.0 (5.0)	3.0 (4.0)	NR	CR
Waterson [42]	2016	United Kingdom	KA/MA	36	35	NR		NR		1	NR		MPP	CR
Matsumoto [7]	2017	Japan	rKA/MA	30	30	75.3 (7.5)	76.1 (7.3)	88.0	90.0	1	12.4 (4.7)	10.4 (4.4)	MPP	CR
Laende [43]	2019	Canada	KA/MA	24	23	64.0 (8.0)	63.0 (7.0)	66.7	73.9	2	6.5 (5.6)	4.9 (4.9)	NR	CR
Seon [49, 50]	2019	South Korea	rKA/MA	30	30	72.0 (5.5)	74.0 (5.2)	83.3	90.0	8	9.7 (6.5)	8.9 (4.0)	MPP	CR
McEwen [44]	2020	Australia	KA/MA	40	40	NR		NR		2	NR		NR	CR
MacDessi [17]	2020	Australia	rKA/MA	70	68	67.4 (13.3)	69.0 (7.5)	63.5	54.8	1	2.8 (9.0)	3.7 (8.1)	NR	PS
Young [45–47]	2020	United States	KA/MA	49	50	72.0 (6.5)	70.0 (7.5)	51.0	52.0	5	1.6 (6.0)	0.8 (6.0)	MPP	CR
Sarzaeem [48]	2021	Iran	KA/MA	32	32	62.9 (6.0)	65.2 (6.8)	43.7	46.9	0.25	NR		NR	NR

Values are expressed as means

*SD* Standard deviation, *F/U* Follow-up, *y* years, *KA* Kinematically aligned, *MA* Mechanically aligned, *rKA* restricted kinematically aligned, *NR* Not reported, *MPP* Medial parapatellar approach, *CR* Cruciate retaining, *PS* Posterior stabilized

#### Primary outcomes

##### Range of motion

Six studies were included in the meta-analysis; four compared MATKA and KATKA [40, 42, 44, 46], and two compared MATKA and rKATKA [7, 49].

**Fig. 3:**
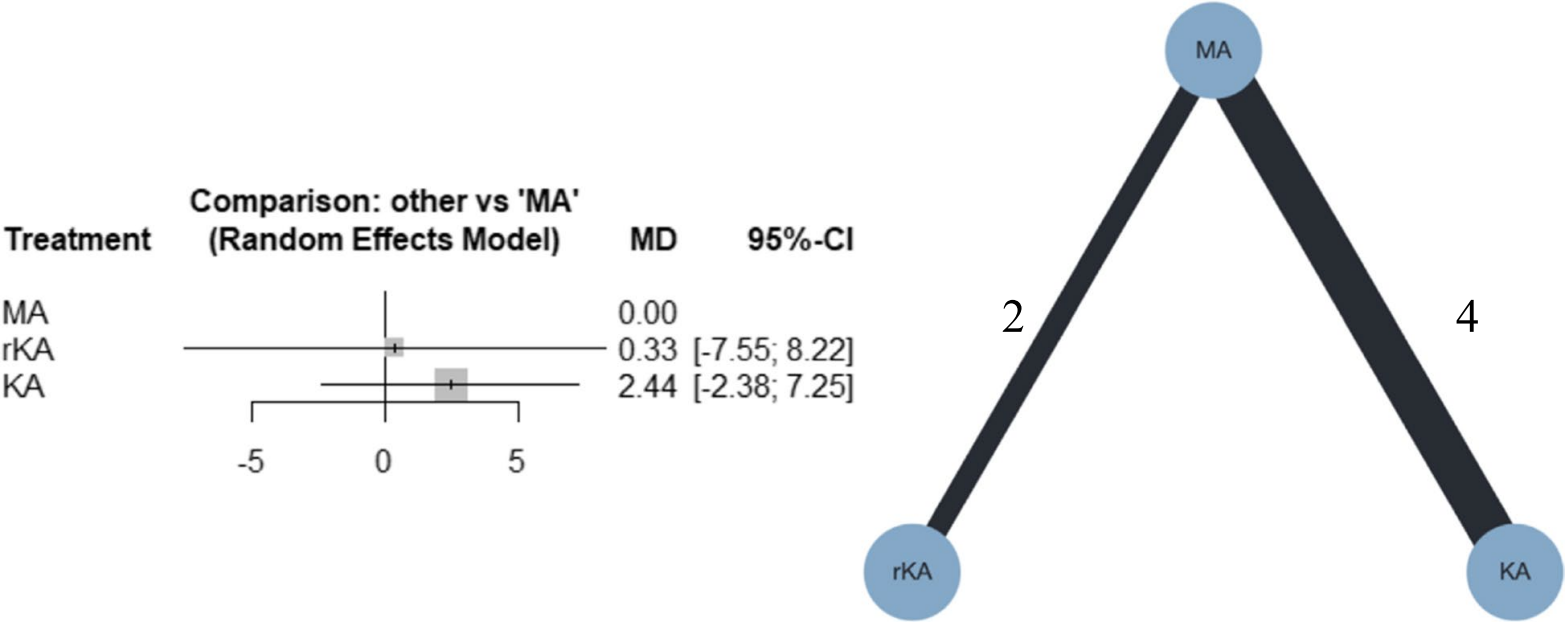
Forest plot and network plot for postoperative ROM. ROM, range of motion; MA, mechanically aligned; KA, kinematically aligned; rKA, restricted kinematically aligned; MD, mean difference; CI, confidence interval

The forest plots for NMA and the network plot are shown in Fig. 3. From the NMA, KATKA and rKATKA might result in little to no difference, compared with MATKA, regarding ROM (MD, 2.44; 95% CI, -2.38–7.25; and MD, 0.33; 95% CI, -7.55–8.22, respectively). Additional file 4a shows that the confidence ratings were very low. Additional file 5a shows the league table. KATKA was ranked first, followed by rKATKA and MATKA, with P-scores of 58%, 49%, and 43%, respectively. I^2^ was 0%, and the *p*-value for global inconsistency was 0.6390.

##### PROMs

**Fig. 4:**
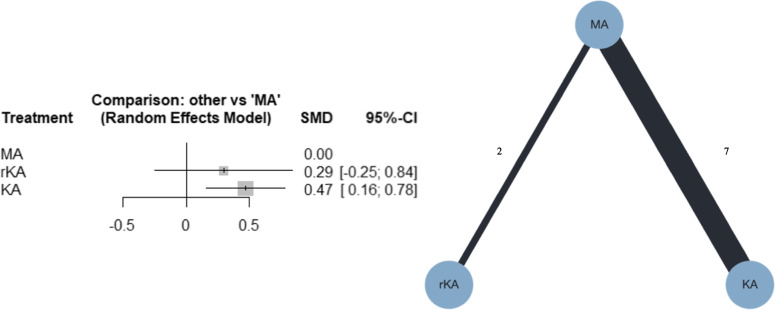
Forest plot and network plot for PROMs. PROMs, patient-reported outcome measures; MA, mechanically aligned; KA, kinematically aligned; rKA, restricted kinematically aligned; SMD, standardized mean difference; CI, confidence interval

Nine studies were included in the meta-analysis; seven compared MATKA and KATKA [40–44, 47, 48], and two compared MATKA and rKATKA [17, 49]. The measured scores differed across studies, and some used multiple scoring systems. As previously mentioned, we pooled scores using WOMAC in four studies [40, 41, 47, 49], OKS in three [43, 44, 48], and KOOS in two [17, 42]. The forest and network plots are shown in Fig. 4. From the NMA, KATKA might result in slightly improved PROMs, compared with MATKA (SMD, 0.47; 95% CI, 0.16–0.78), whereas rKATKA may result in little to no difference, compared with MATKA (SMD, 0.29; 95% CI, -0.26–0.84). Additional file 4b shows that the confidence rating was very low. Additional file 5b shows the league table on PROMs. KATKA ranked first, followed by rKATKA and MATKA, with P-scores of 85%, 57%, and 7%, respectively. I^2^ was 0%, and the *p*-value for global inconsistency was 0.5514.

##### Revision

**Fig. 5:**
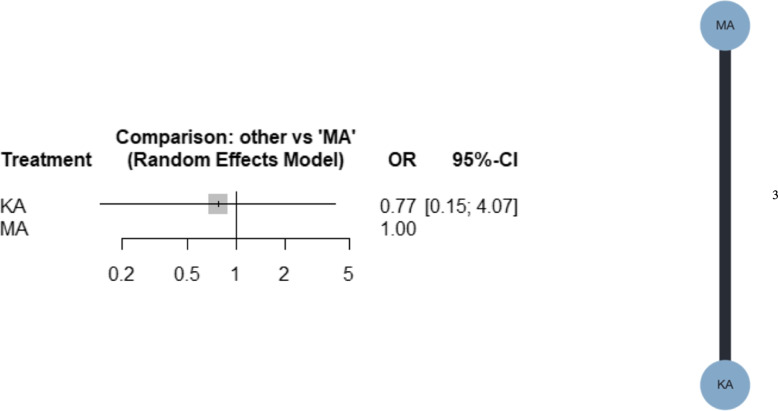
Forest plot and network plot for revision. MA, mechanically aligned; KA, kinematically aligned; rKATKA, restricted kinematically aligned; OR, odds ratio; CI, confidence interval

Three studies were included in the meta-analysis [40, 41, 47]. No study compared MATKA with rKATKA. Forest plots for the NMA and network plots are shown in Fig. 5. From the NMA, KATKA might result in little to no difference compared with MATKA (OR, 0.77; 95% CI, 0.15–4.07). Additional file 4c shows that the confidence rating was very low. Additional file 5c shows the league table of revision. KATKA ranked first, followed by MATKA, with P-scores of 62% and 38%, respectively. I^2^ was 0%, and the *p*-value for global inconsistency was 0.5650.

#### Secondary outcomes

##### Femoral component alignment

**Fig. 6:**
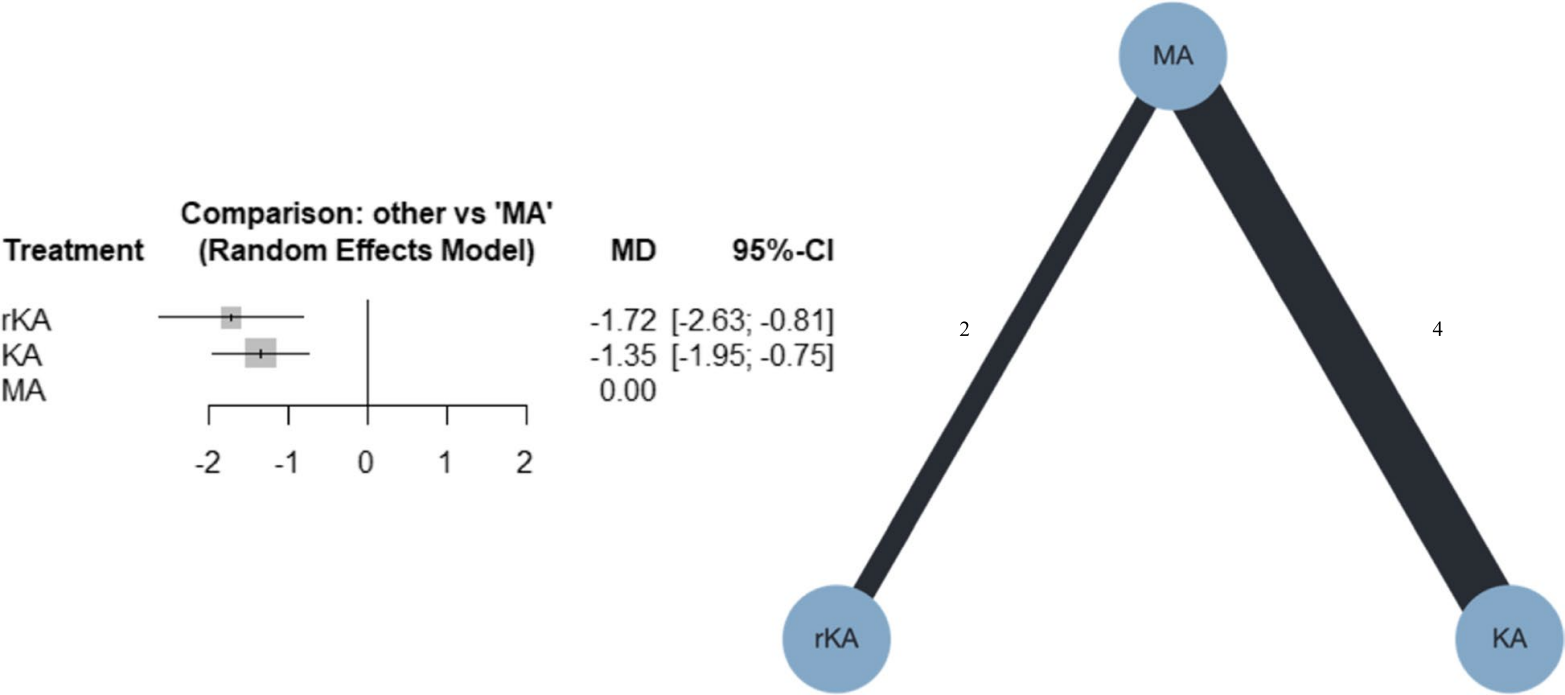
Forest plot and network plot for femoral component alignment. MA, mechanically aligned; KA, kinematically aligned; rKA, restricted kinematically aligned; MD, mean difference; CI, confidence interval

Six studies were included in the meta-analysis; four compared MATKA and KATKA [40, 41, 44, 46], and two compared MATKA and rKATKA [17, 49]. Forest plots for the NMA and network plots are shown in Fig. 6. According to the NMA, compared with MATKA, KATKA and rKATKA might have resulted in a slight valgus (MD, -1.35; 95% CI, -1.95–[-0.75]; and MD, -1.72; 95% CI, -2.63–[-0.81], respectively). Additional file 4d shows that the confidence rating was very low. Additional file 5d shows the league table of femoral component alignment. MATKA ranked first, followed by KATKA and rKATKA, with P-scores of 100%, 37%, and 13%, respectively. I^2^ was 11.8%, and the *p*-value for global inconsistency was 0.3382.

##### Tibial component alignment

**Fig. 7:**
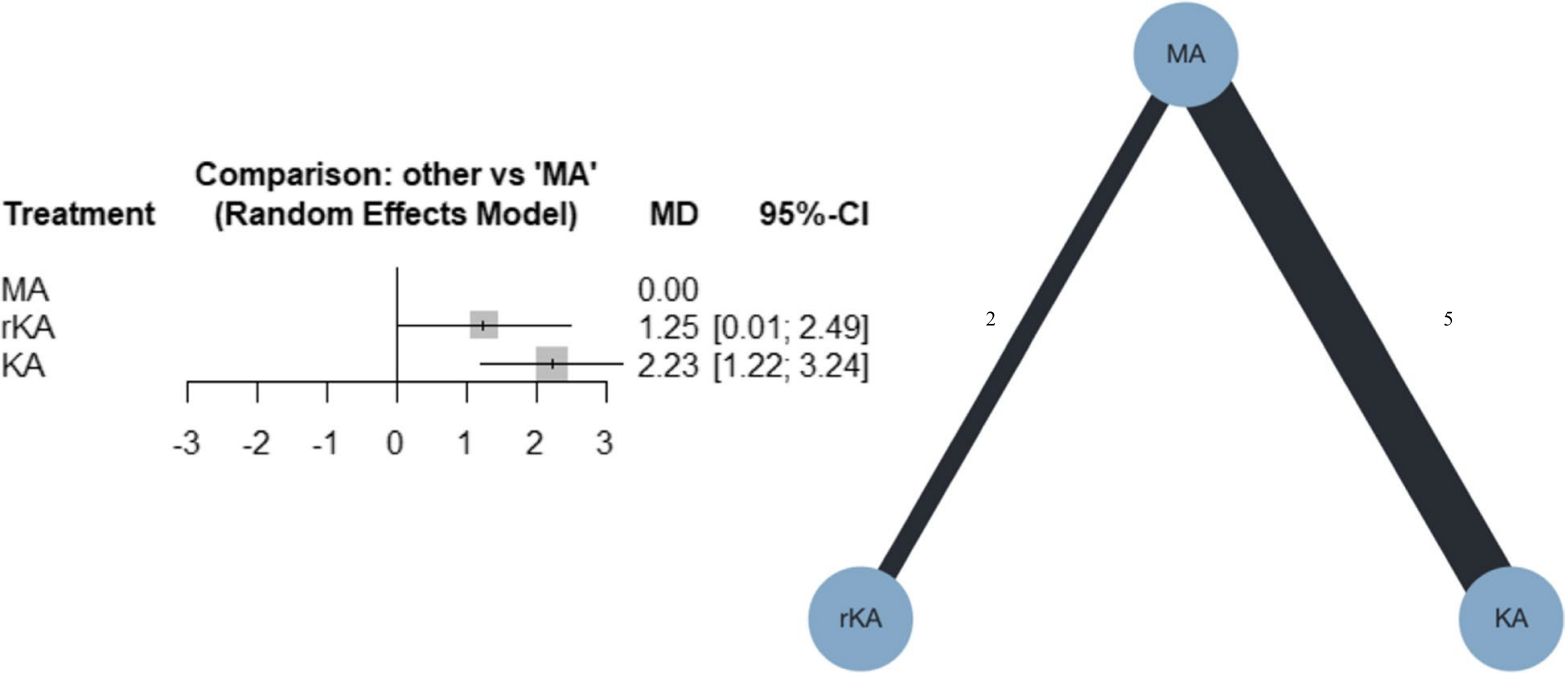
Forest plot and network plot for tibial component alignment. MA, mechanically aligned; KA, kinematically aligned; rKATKA, restricted kinematically aligned; MD, mean difference; CI, confidence interval

Seven studies were included in the meta-analysis; five compared MATKA and KATKA [40, 41, 43, 44, 46], and two compared MATKA and rKATKA [17, 49]. Forest and network plots for the NMA are shown in Fig. 7. From the NMA, compared with MATKA, KATKA, and rKATKA might have resulted in a slight varus (MD, 2.23; 95% CI, 1.22–3.24; and MD, 1.25; 95% CI, 0.01–2.49, respectively). Additional file 4e shows that the confidence rating was very low. Additional file 5e shows the league table of tibial alignment. MATKA ranked first, followed by rKATKA and KATKA, with P-scores of 99%, 45%, and 6%, respectively. I^2^ was 42.9%, and the *p*-value for global inconsistency was 0.1354.

##### Tibial component inclination

**Fig. 8:**
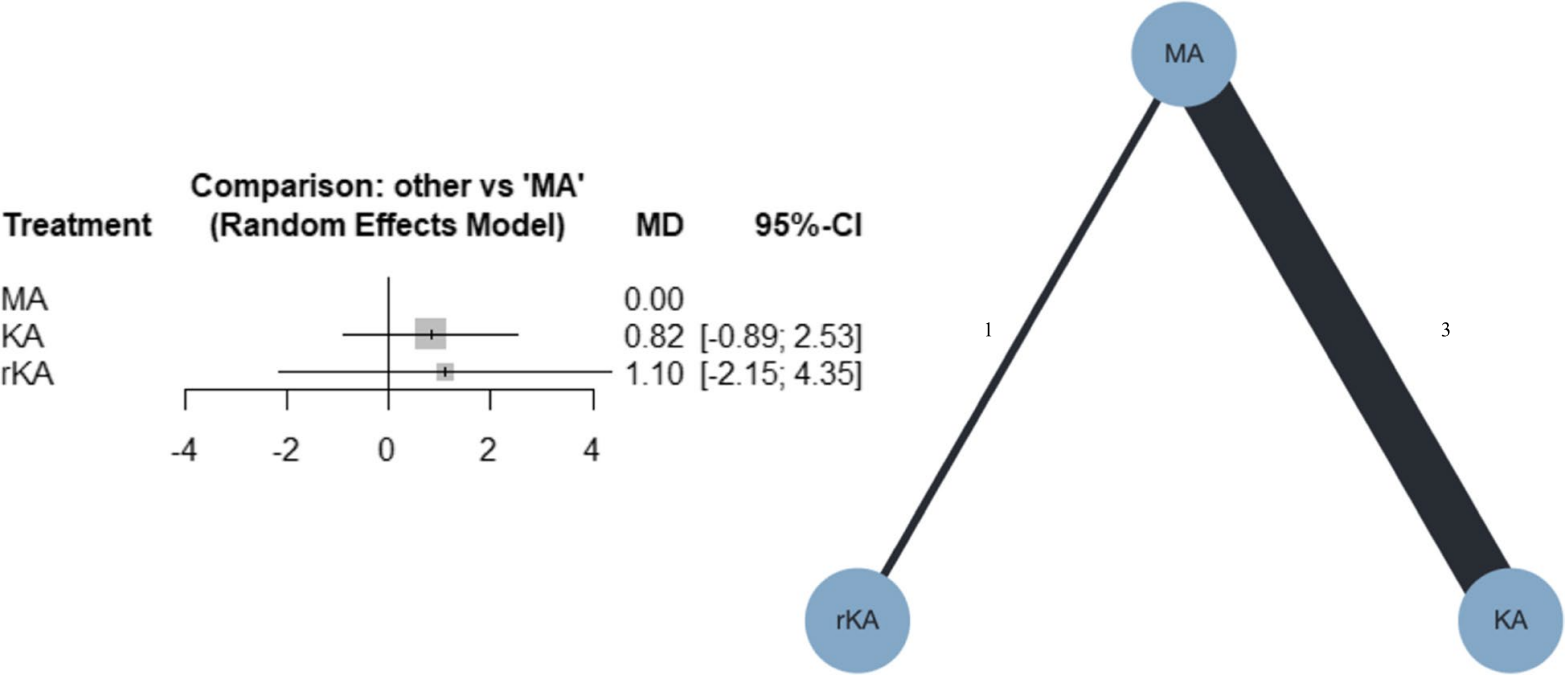
Forest plot and network plot for tibial component inclination. MA, mechanically aligned; KA, kinematically aligned; rKA, restricted kinematically aligned; MD, mean difference; CI, confidence interval

Four studies were included in the meta-analysis; three compared MATKA and KATKA [41, 44, 46], and one compared MATKA and rKATKA [49]. Forest plots for the NMA and network plots are shown in Fig. 8. From the NMA, KATKA and rKATKA might result in little to no difference, compared with MATKA (MD, 0.82; 95% CI, -0.89–2.53; and MD, 1.10; 95% CI, -2.15–4.35, respectively). Additional file 4f shows that the confidence ratings were very low. Additional file 5f shows the league table of inclination of the tibial component. MATKA was ranked first, followed by KATKA and rKATKA, with P-scores of 79%, 37%, and 35%, respectively. I^2^ was 71.1%, and the *p*-value for global inconsistency was 0.0313.

##### HKA

**Fig. 9:**
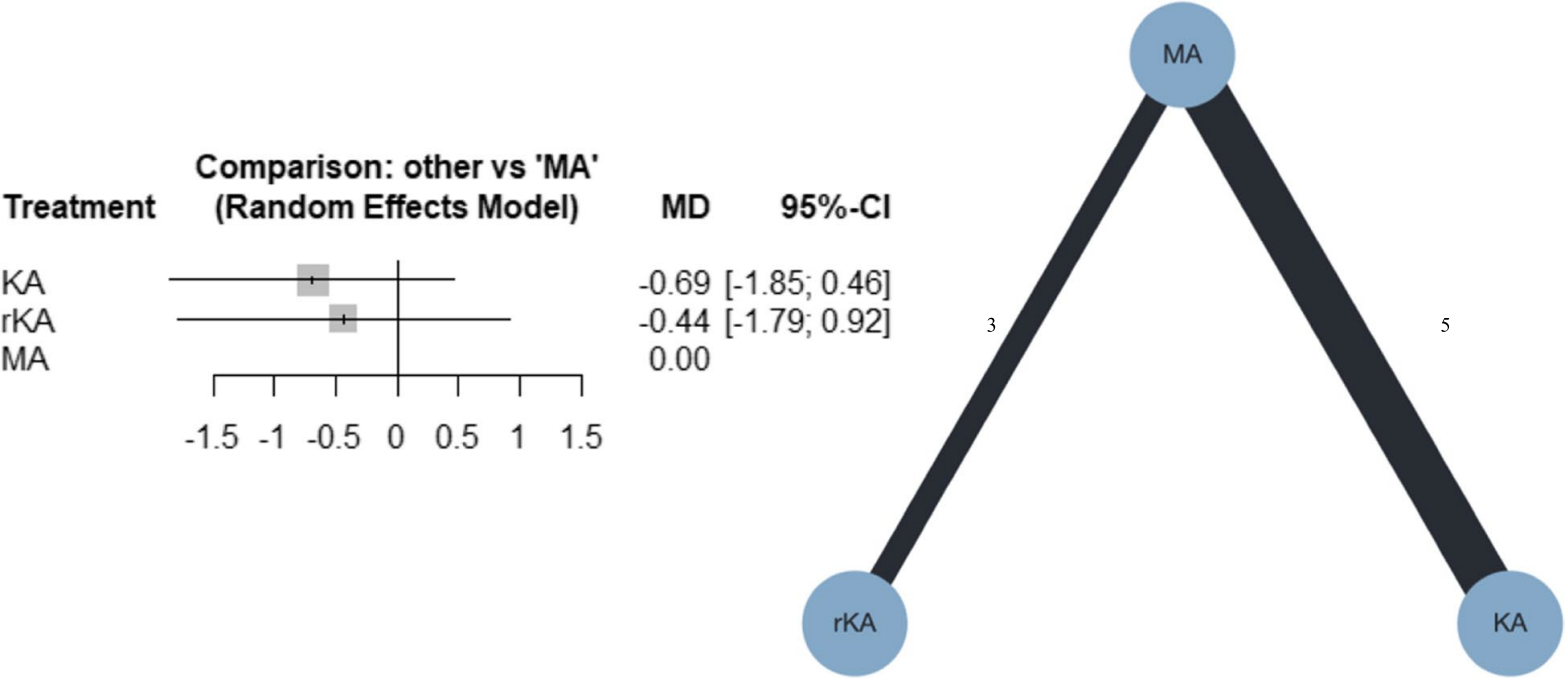
Forest plot and network plot for HKA. HKA, hip-knee-ankle angle; MA, mechanically aligned; KA, kinematically aligned; rKA, restricted kinematically aligned; MD, mean difference; CI, confidence interval

Eight studies were included in the meta-analysis; five compared MATKA and KATKA [40, 41, 43, 44, 46], and three compared MATKA and rKATKA [7, 17, 49]. Forest and network plots for the NMA are shown in Fig. 9. According to the NMA, KATKA and rKATKA might result in little to no difference, compared with the MATKA (MD, -0.69; 95% CI, -1.85–0.46; and MD, -0.44; 95% CI, -1.79–0.92, respectively). Additional file 4g shows that the confidence rating was very low. Additional file 5g shows the league table for HKA. MATKA was ranked first, followed by rKATKA and KATKA, with P-scores of 81%, 44%, and 25%, respectively. I^2^ was 49.0%, and the *p*-value for global inconsistency was 0.0674. The forest and funnel plots for the pairwise analysis of all outcomes are shown in Additional files 6 and 7.

#### All adverse events

In the studies included in our review, three reported adverse events; there were 134 patients in the KATKA group and 135 in the MATKA group [40, 44, 46, 47]. Two patients were infected in each group (1.5%). Two patients in the MATKA group (1.5%) experienced fractures. One patient in the KATKA group (0.7%) and two in the MATKA group (1.5%) had hematomas. Two patients in the KATKA group (1.5%) and one in the MATKA group (0.7%) underwent excision of lateral patella surgery. Four patients in the KATKA group (3.0%) and two in the MATKA group (1.5%) underwent manipulation under anesthesia because of stiffness. One patient in the KATKA group underwent resurfacing of the patella owing to pain (0.7%). One patient underwent MATKA owing to patella dislocation (0.7%).

### Additional analysis

#### Subgroup analysis

Six of the CR insert studies compared MATKA and KATKA [40–43, 46, 48], and two compared MATKA and rKATKA [7, 44]. KATKA might have resulted in slightly improved PROMs, compared with MATKA (SMD, 0.43; 95% CI, 0.10–0.76), and rKATKA might have resulted in little to no difference in TKA, compared with MATKA (SMD, 0.59; 95% CI, -0.41–1.59) (Additional file 8a). Additional file 4h shows that the confidence rating was very low. Additional File 5h shows the league table of PROMs. Overall, rKATKA ranked first, followed by KATKA and MATKA, with *P*-values of 75%, 69%, and 6%, respectively. I^2^ was 0%, and the *p*-value for global inconsistency was 0.4290.

Among the MPP approach studies, two compared MATKA and KATKA [42, 48], and three compared MATKA and rKATKA [7, 17, 49]. KATKA and rKATKA might have resulted in little to no difference in PROMs, compared with MATKA (SMD, 0.14; 95% CI, -0.45–0.73; and SMD, 0.29; 95% CI, -0.25–0.84, respectively) (Additional file 8b). Additional file 4i shows that the confidence rating was very low. Additional file 5i shows the league table. Overall, rKATKA ranked first, followed by KATKA and MATKA, with *P*-values of 75%, 51%, and 23%, respectively. I^2^ was 0%, and the *p*-value for global inconsistency was 0.7723.

#### Sensitivity analysis

We conducted a sensitivity analysis for PROMs after excluding one report with a follow-up period of less than one year [48]. The results demonstrated robustness and validity. The forest plot, confidence rating, and league table are shown in Additional files 4j, 5j, and 8c, respectively.

## Discussion

This NMA of 10 RCTs with a median follow-up time of 1.5 years produced the following findings: First, postoperative ROM and PROMs after KATKA and rKATKA were comparable to those after MATKA. Second, revision rates and adverse events were not reported in rKATKA and were equivalent between MATKA and KATKA. Third, compared with MATKA, postoperative coronal component alignments after KATKA and rKATKA showed slight variation. Finally, the postoperative HKA and tibial component inclination after KATKA and rKATKA were similar to those after MATKA.

The NMA did not show KATKA and rKATKA superiority over MATKA in terms of ROM and PROMs. Our results regarding ROM were consistent with those of recent systematic reviews [15, 19, 21]. Different results have been reported for PROMs, with KATKA being superior [20, 21] or comparable to MATKA [15, 19]. Previous reviews included only head-to-head two-arm studies, limited trials, non-RCTs, or doubly counted participants. We believe that our NMA is methodologically sound and provides rigorous, updated evidence. Furthermore, we synthesized several PROMs with SMD in this study and found that KATKA provided better outcomes than MATKA. However, the small effect size and very low confidence ratings weakened the results [51]. Therefore, we concluded that the clinical superiority of KATKA over MATKA is uncertain. rKATKA is a recently reported technique with a small number of RCTs. In the present review, studies reported as having “anatomical alignment” or KATKA were also included as rKATKA studies if bone resection was restricted to the same degree as that in rKATKA [7, 49, 50]. Despite this, we could only conduct an indirect comparison between KATKA and rKATKA. The main and additional analyses showed comparable PROMs for rKATKA compared with the other two procedures.

Our research did not find any advantages of rKATKA or disadvantages of KATKA related to revision surgery. The principle of KATKA is to recreate the pre-arthritic articular surface of the patient’s native knee using TKA components [5]. However, individuals' lower limb alignment and joint line obliquity vary [52]. Furthermore, the pre-arthritic articular surface differed from that of severe to end-stage KOA, and the tibia varus progressed by approximately 10° with advanced osteoarthritis [53]. Therefore, there are concerns that KATKA increases loading to the prosthetic knee owing to excessive varus alignment, subsequent implant migration, and revision surgery. Such concerns are reduced in rKATKA. In this study, the median follow-up period was short, and only a few reports were available, causing the insignificant difference in revision outcomes between KATKA and MATKA. Furthermore, revision and adverse events have not been described for rKATKA. However, the results may not be sufficient to dismiss the potential benefits of these two new methods. Functional joint line orientation is another factor affecting the prosthetic knee's dynamic loading. In fact, despite a range in alignment, the joint line in a standing position tends to remain parallel to the ground in healthy asymptomatic knees, even after KATKA and rKATKA [7, 54, 55]. Therefore, physiological articular surface reconstruction may have a favorable effect on the overall load of the prosthetic joint. Long-term results are still lacking, with only two studies having a follow-up period of more than five years, the longest of which was eight years [47, 49]. Further long-term follow-up studies are required to clarify this issue.

KATKA and rKATKA showed a slight valgus femoral component and a slight varus tibial component, compared with MATKA; however, the difference between the postoperative component alignments of KATKA and rKATKA was unclear. Furthermore, HKA was similar among the three methods. In this review, four studies showed a preoperative lower limb coronal alignment of less than 3° varus [17, 39–41, 45–47]. Only one study reported > 10° varus, and the intervention was rKATKA [7]. Therefore, the similarity in coronal alignment between KATKA and rKATKA may be owing to the limited number of studies with severe varus deformity. Thus, further subgroup analysis of the varus deformity group was impossible, and the external validity for these patients was low. Additional RCTs are needed to confirm the selection of the most appropriate method in patients with severe deformity.

### Strengths and limitations

Some limitations should be considered when interpreting our findings.

First, the number of studies included in the NMA was small, which may have led to bias and heterogeneity. Second, the KATKA and rKATKA procedures have been gradually adopted in recent years; thus, many included studies have a short follow-up period. Third, although we performed an additional analysis, there might still be clinical heterogeneity, including different surgical techniques, prosthesis types, rehabilitation training, and preoperative limb alignments. However, this is the first NMA to compare the efficacy of the three different surgical techniques in TKA. The strength of this study lies in the indirect comparison between KATKA and rKATKA because there are no direct comparisons. Indirect comparisons can provide useful information regarding the relative efficacy of competing interventions. Furthermore, we carefully and rigorously designed the screening, extraction, and evaluation of confidence in the evidence from NMA using CINeMA, according to the Cochrane Handbook. Further large-scale, long-term follow-up period studies of patients with severe varus deformities of the knee and completion of the ongoing trial are needed to establish the safety and evaluate the effectiveness of the surgical techniques presented (Additional file 3).

## Conclusions

In conclusion, KATKA and rKATKA are acceptable methods in short- to mid-term follow-up periods. However, long-term clinical outcomes are still lacking and the revision risk in rKATKA has not been reported. Therefore, surgeons should carefully choose the surgical procedure and further trials with long-term follow-up are needed to evaluate the efficacy, safety, and subsequent revision risk.

## Supplementary Information

**Additional file 1.** PRISMA NMA Checklist of Items to Include When Reporting A Systematic Review Involving a Network Meta-analysis.

**Additional file 2.** Search strategies.

**Additional file 3.** Reasons for exclusion of seven reports.

**Additional file 4.** Assessing confidence in the results of a network meta-analysis.

**Additional file 5.** League tables.

**Additional file 6.** Forest plot for the pairwise analysis.

**Additional file 7.** Funnel plots.

**Additional file 8.** Forest plot for PROMs in additional analysis.

## Data Availability

The datasets supporting the conclusions of this article are included within the article.

## Abbreviations

KOA: Knee osteoarthritis
TKA: Total knee arthroplasty
MATKA: Mechanically aligned total knee arthroplasty
KATKA: Kinematically aligned total knee arthroplasty
rKATKA: Restricted kinematically aligned total knee arthroplasty
NMA: Network meta-analysis
RCTs: Randomized controlled trials
OR: Odds ratio
CI: Confidence intervals
MD: Mean difference
SMD: Standardized mean difference
PROMs: Patient-reported outcome measures
WOMAC: Western Ontario and McMaster Universities osteoarthritis index scores
KOOS: Knee Injury and Osteoarthritis Outcome Score
OKS: Oxford knee score
ROB: Risk of bias
CR: Cruciate retaining
PS: Posterior stabilized
MPP: Medial parapatellar approach
