# Supplementary material for: Postoperative clinical outcomes for kinematically, restricted kinematically, or mechanically aligned total knee arthroplasty: a systematic review and network meta-analysis of randomized controlled trials
Source: BMC Musculoskelet Disord. 2023 Apr 24;24:322. doi: 10.1186/s12891-023-06448-0 (PMC10124064; doi:10.1186/s12891-023-06448-0)
Supplement: Supplementary file 2 — Additional file 2. Search strategies. [file 12891_2023_6448_MOESM2_ESM.docx]

**Additional file 2. Search strategies**

| **CENTRAL search strategy (searched on August 20, 2022)** | |
| --- | --- |
| #1: | MeSH descriptor: [arthroplasty, replacement, knee] explode all trees |
| #2: | knee* near (replac* or arthroplast*) |
| #3: | #1 or #2 |
| #4: | kinematic*: ti, ab, kw |
| #5: | anatomic*: ti, ab, kw |
| #6: | #4 or #5 |
| #7: | #3 and #6 |
| **MEDLINE search strategy (searched on August 20, 2022)** | |
| #1: | arthroplasty, replacement, knee [mh] |
| #2: | knee [tiab] AND (replac* [tiab] OR arthroplast* [tiab]) |
| #3: | #1 OR #2 |
| #4: | kinematic* [tiab] |
| #5: | anatomic* [tiab] |
| #6: | #4 OR #5 |
| #7: | #3 OR #6 |
| #8: | randomized controlled trial [pt] |
| #9: | controlled clinical trial [pt] |
| #10: | randomized [tiab] |
| #11: | placebo [tiab] |
| #12: | drug therapy [sh] |
| #13: | randomly [tiab] |
| #14: | trial [tiab] |
| #15: | groups [tiab] |
| #16: | #8 OR #9 OR #10 OR #11 OR #12 OR #13 OR #14 OR #15 |
| #17: | animals [mh] NOT humans [mh] |
| #18: | #16 NOT #17 |
| #19: | #7 AND #18 |
| **EMBASE (via ProQuest Dialog) search strategy (searched on August 20, 2022)** | |
| S1: | EMB.EXACT.EXPLODE("knee arthroplasty") |
| S2: | (AB("knee") OR TI("knee")) AND (AB("replac*") OR TI("replac*") OR AB("arthroplast*") OR TI("arthroplast*")) |
| S3: | S1 OR S2 |
| S4: | EMB.EXACT.EXPLODE("kinematic") |
| S5: | AB("kinematic*") OR TI("kinematic*") |
| S6: | S4 OR S5 |
| S7: | (AB [random*] OR TI [random*]) OR (AB [clinical NEAR/1 trial*] OR TI [clinical NEAR/1 trial*]) OR (EMB.EXACT ["health care quality"]) |
| S8: | S3 AND S6 AND S7 |
| **ICTRP search strategy (searched on August 20, 2022)**  Advanced search | |
| #1: | Title: knee |
| #2: | Conditions: (replacement OR arthroplasty) |
| #3: | Kinematic* |
| #4: | #1 AND #2 AND #3  Recruitment status: is ALL. |
| **ClinicalTrials.gov search strategy (searched on August 20, 2022)**  Advanced search | |
| ((knee replacement OR knee arthroplasty) AND kinematic) | |

ICTRP, International Clinical Trials Registry Platform
