# Supplementary material for: Postoperative clinical outcomes for kinematically, restricted kinematically, or mechanically aligned total knee arthroplasty: a systematic review and network meta-analysis of randomized controlled trials
Source: BMC Musculoskelet Disord. 2023 Apr 24;24:322. doi: 10.1186/s12891-023-06448-0 (PMC10124064; doi:10.1186/s12891-023-06448-0)
Supplement: Supplementary file 3 — Additional file 3. Reasons for exclusion of seven reports. [file 12891_2023_6448_MOESM3_ESM.docx]

**Additional file 3. Reasons for exclusion of seven reports**

| Title | First author | Year | Exclusion Reasons | Digital Object Identifier (DOI) |
| --- | --- | --- | --- | --- |
| A Prospective Randomized Controlled Trial Evaluating Patient Specific Total Knee Arthroplasty Using Kinematic Alignment Versus Ligament-balanced, Mechanical Alignment | Patrick Sadoghi | Ongoing | Ongoing study | NCT04436211 |
| Kinematic Versus Mechanical Alignment in Total Knee Replacement: a Randomized Double-blinded Controlled Study | Max Ettinger | Ongoing | Ongoing study | NCT04384913 |
| A multi-centre, prospective randomised controlled trial to compare modified kinematically aligned total knee arthroplasty using iTKR™ Software with mechanically aligned total knee arthroplasty | Simon Coffey | Ongoing | Ongoing study | ACTRN12618001376235 |

| A Prospective, Randomized, Single-Blinded, Multi-Center, Clinical Trial Comparing Kinematic Alignment vs. Mechanical Alignment Using Medially-Stabilized Knee (GMK Sphere) And Patient-Specific Cutting Guides (MyKnee) For Total Knee Arthroplasty | Rena Mandino | Ongoing | Ongoing study | NCT05461638 |
| --- | --- | --- | --- | --- |
| A comparison of mechanical and kinematic alignment principles in total knee joint replacement using a medial pivot design prosthesis | Mike van Niekirk | Ongoing | Ongoing study | ACTRN12620000892910p |
| A prospective double-blinded randomised control trial comparing robotic arm-assisted functionally aligned total knee arthroplasty versus robotic arm-assisted mechanically aligned total knee arthroplasty | Babar Kayani | Ongoing | Ongoing study | NCT04092153. |
| Comparison of clinical and biomechanical outcomes between the kinematic and mechanical alignment methods in total knee arthroplasty: Protocol for a multicenter randomized controlled trial | Yoshinori Takashima | Ongoing | Ongoing study | UMIN #000026895 |
