# Supplementary material for: Postoperative clinical outcomes for kinematically, restricted kinematically, or mechanically aligned total knee arthroplasty: a systematic review and network meta-analysis of randomized controlled trials
Source: BMC Musculoskelet Disord. 2023 Apr 24;24:322. doi: 10.1186/s12891-023-06448-0 (PMC10124064; doi:10.1186/s12891-023-06448-0)
Supplement: Supplementary file 4 — Additional file 4. Assessing confidence in the results of a network meta-analysis. [file 12891_2023_6448_MOESM4_ESM.docx]

**Additional file 4. Assessing confidence in the results of a network meta-analysis**

MA, mechanically aligned; KA, kinematically aligned; rKA, restricted kinematically aligned; PROMs, patient-reported outcome measures; HKA, hip-knee-ankle angle; CR, cruciate retaining; MPP, medial parapatellar

**4a Range of motion**

| **Comparison** | **Number of studies** | **Within-study bias** | **Reporting bias** | **Indirectness** | **Imprecision** | **Heterogeneity** | **Incoherence** | **Confidence rating** |
| --- | --- | --- | --- | --- | --- | --- | --- | --- |
| **KA:MA** | 4 | Some concerns | Some concerns | Low risk | Some concerns | Some concerns | Major concerns | Very low |
| **MA:rKA** | 2 | Some concerns | Low risk | Low risk | Major concerns | No concerns | Major concerns | Very low |
| **KA:rKA** | 0 | Some concerns | Low risk | Low risk | Some concerns | Some concerns | Major concerns | Very low |

**4b PROMs**

| **Comparison** | **Number of studies** | **Within-study bias** | **Reporting bias** | **Indirectness** | **Imprecision** | **Heterogeneity** | **Incoherence** | **Confidence rating** |
| --- | --- | --- | --- | --- | --- | --- | --- | --- |
| **KA:MA** | 7 | Some concerns | Some concerns | No concerns | No concerns | Some concerns | Major concerns | Very low |
| **MA:rKA** | 2 | Some concerns | Some concerns | No concerns | Some concerns | Some concerns | Major concerns | Very low |
| **KA:rKA** | 0 | Some concerns | Some concerns | No concerns | Major concerns | No concerns | Major concerns | Very low |

**4c Revision**

| **Comparison** | **Number of studies** | **Within-study bias** | **Reporting bias** | **Indirectness** | **Imprecision** | **Heterogeneity** | **Incoherence** | **Confidence rating** |
| --- | --- | --- | --- | --- | --- | --- | --- | --- |
| **KA:MA** | 3 | Some concerns | Some concerns | No concerns | No concerns | Some concerns | Major concerns | Very low |

**4d Femoral component alignment**

| **Comparison** | **Number of studies** | **Within-study bias** | **Reporting bias** | **Indirectness** | **Imprecision** | **Heterogeneity** | **Incoherence** | **Confidence rating** |
| --- | --- | --- | --- | --- | --- | --- | --- | --- |
| **KA:MA** | 4 | Some concerns | Some concerns | Some concerns | No concerns | No concerns | Major concerns | Very low |
| **MA:rKA** | 2 | Some concerns | Some concerns | Some concerns | No concerns | Some concerns | Major concerns | Very low |
| **KA:rKA** | 0 | Some concerns | Some concerns | Some concerns | No concerns | Major concerns | Major concerns | Very low |

**4e Tibial component alignment**

| **Comparison** | **Number of studies** | **Within-study bias** | **Reporting bias** | **Indirectness** | **Imprecision** | **Heterogeneity** | **Incoherence** | **Confidence rating** |
| --- | --- | --- | --- | --- | --- | --- | --- | --- |
| **KA:MA** | 5 | Some concerns | Some concerns | Some concerns | No concerns | Major concerns | Major concerns | Very low |
| **MA:rKA** | 2 | Some concerns | Low risk | Some concerns | Some concerns | Some concerns | Major concerns | Very low |
| **KA:rKA** | 0 | Some concerns | Low risk | Some concerns | Some concerns | Some concerns | Major concerns | Very low |

**4f Tibial component inclination**

| **Comparison** | **Number of studies** | **Within-study bias** | **Reporting bias** | **Indirectness** | **Imprecision** | **Heterogeneity** | **Incoherence** | **Confidence rating** |
| --- | --- | --- | --- | --- | --- | --- | --- | --- |
| **KA:MA** | 3 | Some concerns | Low risk | Some concerns | Some concerns | Some concerns | Major concerns | Very low |
| **MA:rKA** | 1 | Some concerns | Some concerns | Some concerns | Major concerns | No concerns | Major concerns | Very low |
| **KA:rKA** | 0 | Some concerns | Some concerns | Some concerns | Major concerns | No concerns | Major concerns | Very low |

**4g HKA**

| **Comparison** | **Number of studies** | **Within-study bias** | **Reporting bias** | **Indirectness** | **Imprecision** | **Heterogeneity** | **Incoherence** | **Confidence rating** |
| --- | --- | --- | --- | --- | --- | --- | --- | --- |
| **KA:MA** | 5 | Some concerns | Low risk | Some concerns | Some concerns | Some concerns | Major concerns | Very low |
| **MA:rKA** | 3 | Some concerns | Low risk | Some concerns | Major concerns | No concerns | Major concerns | Very low |
| **KA:rKA** | 0 | Some concerns | Some concerns | Some concerns | Major concerns | No concerns | Major concerns | Very low |

**4h PROMs in CR insert studies**

| **Comparison** | **Number of studies** | **Within-study bias** | **Reporting bias** | **Indirectness** | **Imprecision** | **Heterogeneity** | **Incoherence** | **Confidence rating** |
| --- | --- | --- | --- | --- | --- | --- | --- | --- |
| **KA:MA** | 6 | Some concerns | Some concerns | No concerns | No concerns | Major concerns | Major concerns | Very low |
| **MA:rKA** | 1 | Some concerns | Some concerns | No concerns | Some concerns | Some concerns | Major concerns | Very low |
| **KA:rKA** | 0 | Some concerns | Some concerns | No concerns | Major concerns | No concerns | Major concerns | Very low |

**4i PROMs in MPP approach studies**

| **Comparison** | **Number of studies** | **Within-study bias** | **Reporting bias** | **Indirectness** | **Imprecision** | **Heterogeneity** | **Incoherence** | **Confidence rating** |
| --- | --- | --- | --- | --- | --- | --- | --- | --- |
| **KA:MA** | 2 | Some concerns | Some concerns | No concerns | Some concerns | Some concerns | Major concerns | Very low |
| **MA:rKA** | 2 | Some concerns | Some concerns | No concerns | No concerns | Major concerns | Major concerns | Very low |
| **KA:rKA** | 0 | Some concerns | Some concerns | No concerns | Some concerns | Some concerns | Major concerns | Very low |

4j PROMs more than one-year follow-up studies

| **Comparison** | **Number of studies** | **Within-study bias** | **Reporting bias** | **Indirectness** | **Imprecision** | **Heterogeneity** | **Incoherence** | **Confidence rating** |
| --- | --- | --- | --- | --- | --- | --- | --- | --- |
| **KA:MA** | 6 | Some concerns | Some concerns | No concerns | No concerns | Major concerns | Major concerns | Very low |
| **MA:rKA** | 2 | Some concerns | Some concerns | No concerns | Some concerns | Some concerns | Major concerns | Very low |
| **KA:rKA** | 0 | Some concerns | Some concerns | No concerns | Major concerns | No concerns | Major concerns | Very low |
