# Supplementary material for: Postoperative clinical outcomes for kinematically, restricted kinematically, or mechanically aligned total knee arthroplasty: a systematic review and network meta-analysis of randomized controlled trials
Source: BMC Musculoskelet Disord. 2023 Apr 24;24:322. doi: 10.1186/s12891-023-06448-0 (PMC10124064; doi:10.1186/s12891-023-06448-0)
Supplement: Supplementary file 5 — Additional file 5. League tables. [file 12891_2023_6448_MOESM5_ESM.docx]

**Additional file 5. League tables**

Treatments are ranked from best to worst along the leading diagonal. Above the leading the diagonal are estimates from pairwise meta-analyses, below the leading diagonal are estimates from network meta-analyses.

MA, mechanically aligned; KA, kinematically aligned; rKA, restricted kinematically aligned; PROMs, patient-reported outcome measures; CR, cruciate retaining; MPP, medial parapatellar

**5a Range of motion**

| KA | 2.44 (-2.48–7.25) |  |
| --- | --- | --- |
| 2.44 (-2.38–7.25) | MA | -0.33 (-8.22–7.55) |
| 2.10 (-7.14­11.35) | -0.33 (-8.22­7.55) | rKA |

**5b PROMs**

| KA | 0.47 (0.16–0.78) |  |
| --- | --- | --- |
| 0.47 (0.16–0.78) | MA | -0.29 (-0.84–0.24) |
| 0.17 (-0.45–0.80) | -0.29 (-0.84­0.25) | rKA |

**5c Revision**

| KA | 0.77 (0.15–4.07) |
| --- | --- |
| 0.77 (0.15–4.07) | MA |

**5d Femoral component alignment**

| KA | -1.35 (-1.95–-0.75) |  |
| --- | --- | --- |
| -1.35 (-1.95–-0.75) | MA | 1.72 (0.81–2.63) |
| 0.37 (-0.72–1.46) | 1.72 (0.81­2.63) | rKA |

**5e Tibial component alignment**

| KA | 2.23 (0.01–3.24) |  |
| --- | --- | --- |
| 2.23 (1.22–3.24) | MA | -1.25 (-2.49–-0.01) |
| 0.98 (-0.62–2.59) | -1.25 (-2.49­-0.01) | rKA |

**5f Tibial component inclination**

| KA | 0.82 (-0.89–2.53) |  |
| --- | --- | --- |
| 0.82 (-0.89–2.53) | MA | -1.1 (-4.35–2.15) |
| -0.28 (-3.96–3.34) | -1.1 (-4.35­2.15) | rKA |

**5g HKA**

| KA | -0.69 (-1.85–0.46) |  |
| --- | --- | --- |
| -0.69 (-1.85–0.46) | MA | 0.44 (-0.92–1.79) |
| -0.26 (-2.04–1.52) | 0.44 (-0.92­1.79) | rKA |

**5h PROMs in CR insert studies**

| KA | 0.43 (0.10–0.76) |  |
| --- | --- | --- |
| 0.43 (0.10–0.76) | MA | -0.59 (-1.59–0.41) |
| -0.16 (-1.21–0.89) | -0.59 (-1.59­0.41) | rKA |

**5i PROMs in MPP approach studies**

| KA | 0.14 (-0.45–0.73) |  |
| --- | --- | --- |
| 0.14 (-0.45–0.73) | MA | -0.29 (-0.84–0.25) |
| -0.16 (-0.96–0.65) | -0.29 (-0.84­0.25) | rKA |

5j PROMs more than one-year follow-up studies

| KA | 0.43 (0.10–0.76) |  |
| --- | --- | --- |
| 0.43 (0.10–0.76) | MA | -0.29 (-0.84–0.25) |
| 0.14 (-0.50–0.77) | -0.29 (-0.84­0.25) | rKA |
