# Supplementary material for: Postoperative clinical outcomes for kinematically, restricted kinematically, or mechanically aligned total knee arthroplasty: a systematic review and network meta-analysis of randomized controlled trials
Source: BMC Musculoskelet Disord. 2023 Apr 24;24:322. doi: 10.1186/s12891-023-06448-0 (PMC10124064; doi:10.1186/s12891-023-06448-0)
Supplement: Supplementary file 6 — Additional file 6. Forest plot for the pairwise analysis. [file 12891_2023_6448_MOESM6_ESM.docx]

**Additional file 6. Forest plot for the pairwise analysis**

ROM, range of motion; MA, mechanically aligned; KA, kinematically aligned; rKA, restricted kinematically aligned; PROMs, patient-reported outcome measures; HKA, hip-knee-ankle angle; CR, cruciate retaining; MPP, medial parapatellar

**6a ROM**

(a) MA vs KA, (b) MA vs rKA

(a)


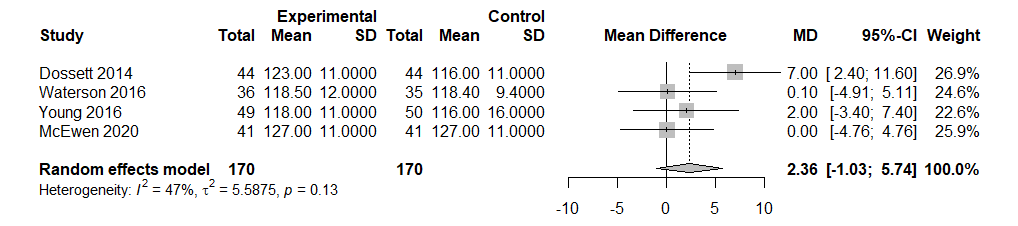


(b)


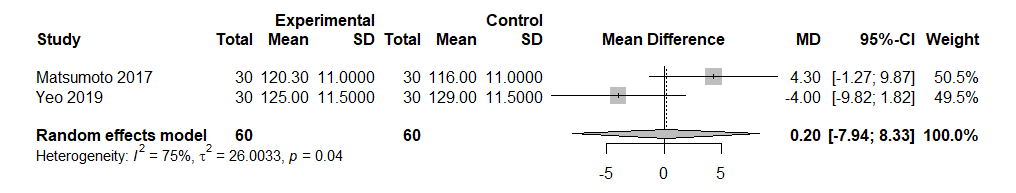


**6b PROMs**

(a) MA vs KA, (b) MA vs rKA


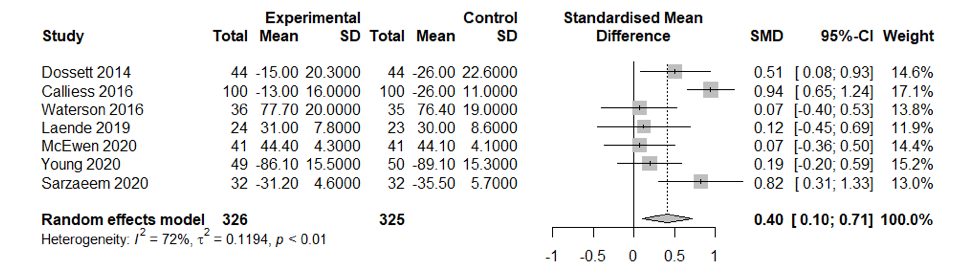
(a)


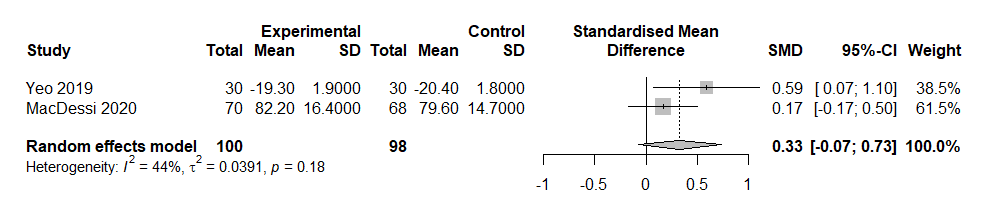
(b)


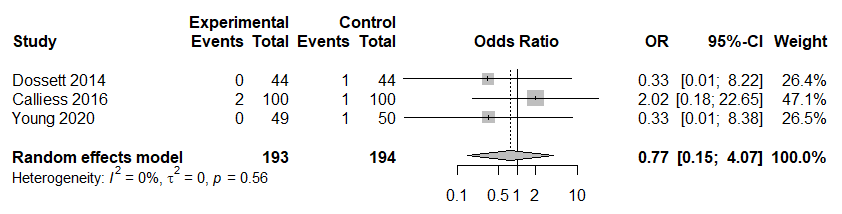


**6c Revision**

**6d Femoral component alignment**

(a) MA vs KA, (b) MA vs rKA

(a)


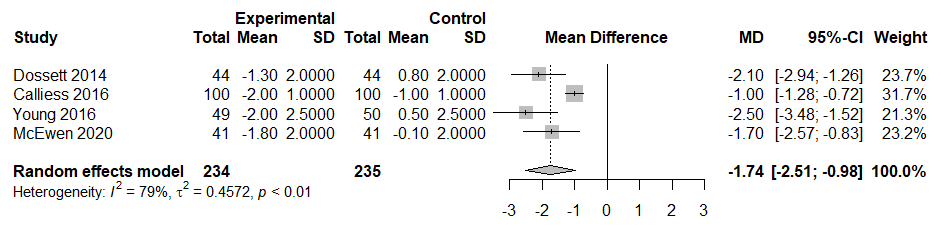


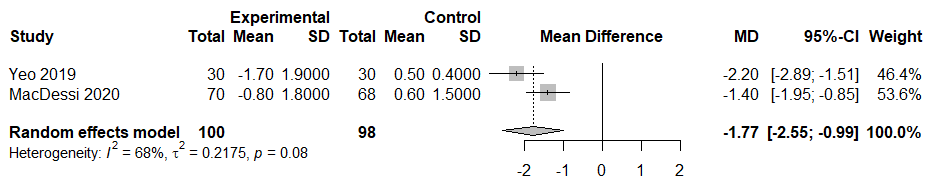
(b)

**6e Tibial component alignment**

(a) MA vs KA, (b) MA vs rKA


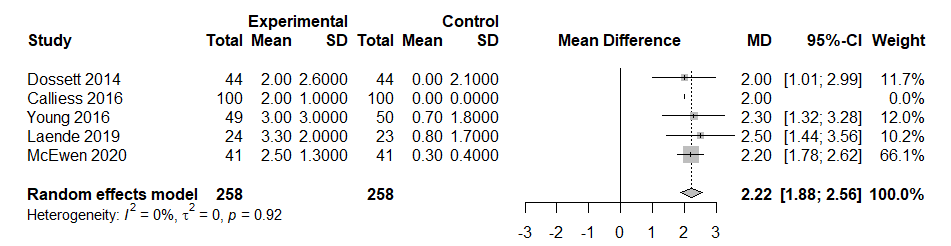
(a)


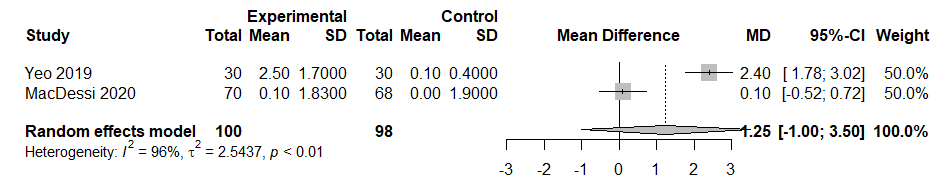
(b)

**6f Tibial component inclination**


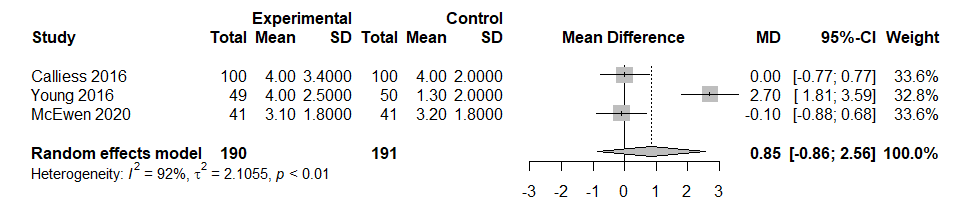
(a) MA vs KA, (b) MA vs rKA

(a)


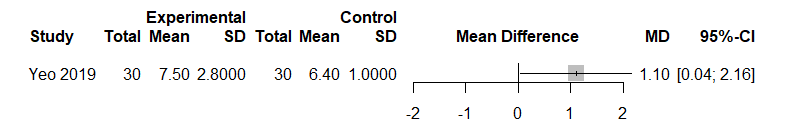
(b)

**6g HKA**

(a) MA vs KA, (b) MA vs rKA


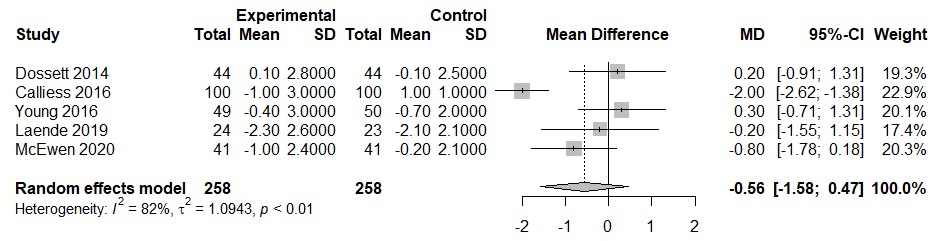
(a)


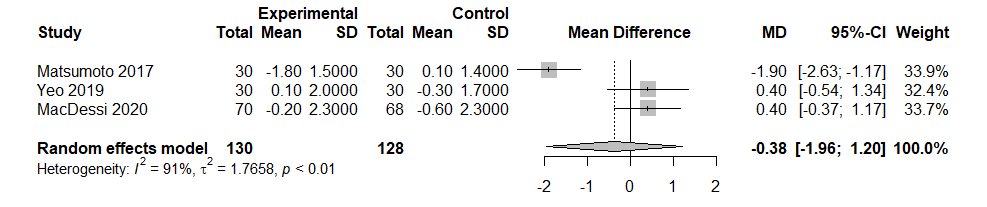
(b)
