# Supplementary material for: Postoperative clinical outcomes for kinematically, restricted kinematically, or mechanically aligned total knee arthroplasty: a systematic review and network meta-analysis of randomized controlled trials
Source: BMC Musculoskelet Disord. 2023 Apr 24;24:322. doi: 10.1186/s12891-023-06448-0 (PMC10124064; doi:10.1186/s12891-023-06448-0)

**Additional file 7. Funnel plots**

ROM, range of motion; MA, mechanically aligned; KA, kinematically aligned; rKA, restricted kinematically aligned; PROMs, patient-reported outcome measures; HKA, hip-knee-ankle angle; CR, cruciate retaining; MPP, medial parapatellar

**7a ROM**

(a) MA vs KA, (b) MA vs rKA

(a) (b)


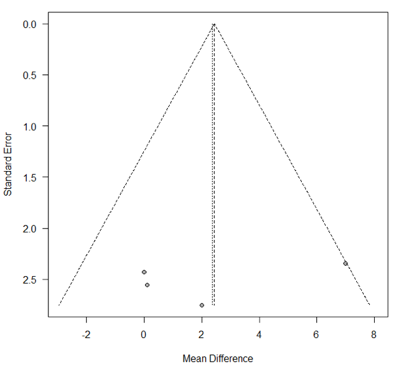

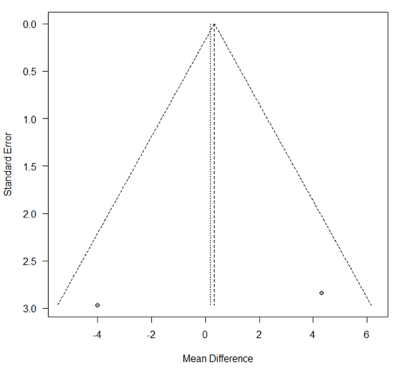


**7b PROMs**

(a) MA vs KA, (b) MA vs rKA

(a) (b)


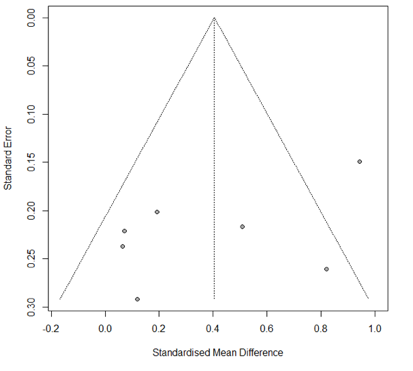

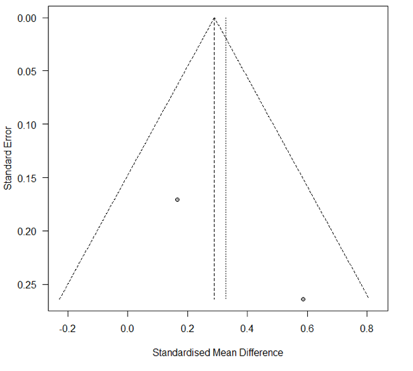


**7c Revision**

MA vs KA


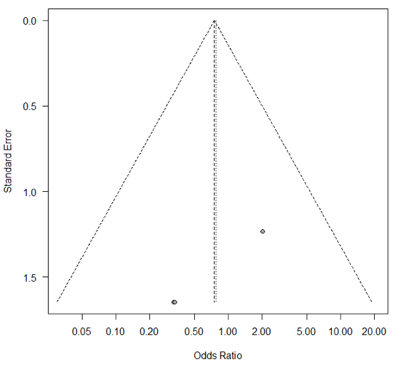


**7d Femoral component alignment**

(a) MA vs KA, (b) MA vs rKA

(a) (b)


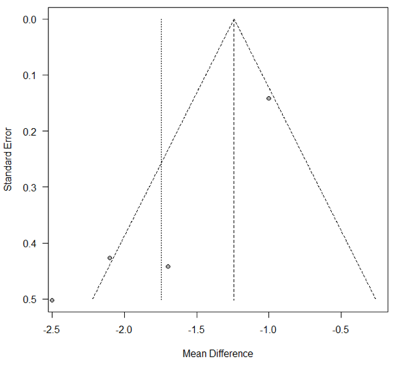

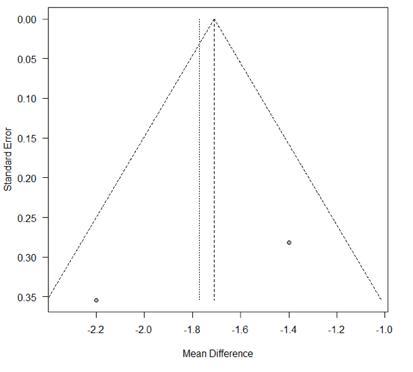


**7e Tibial component alignment**

(a) MA vs KA, (b) MA vs rKA

(a) (b)


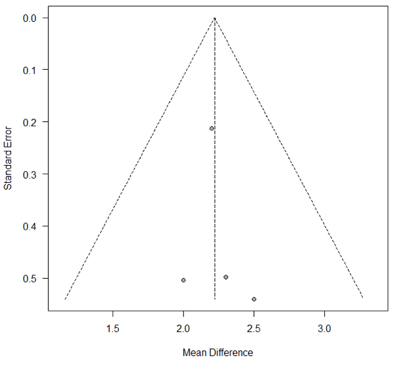
**
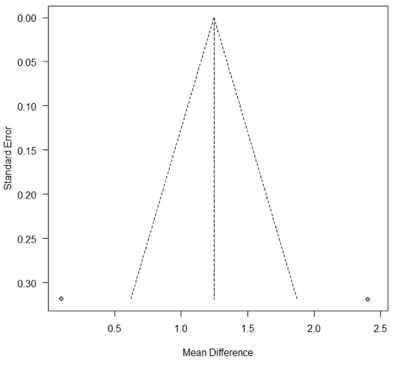
**

**7f Tibial component inclination**

(a) MA vs KA, (b) MA vs rKA

(a) (b)


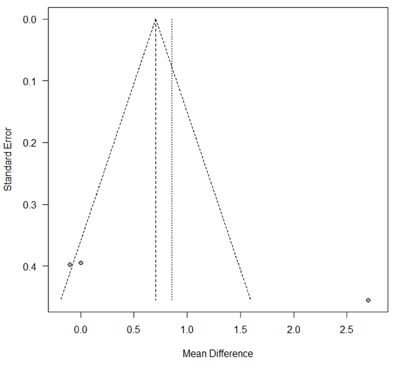

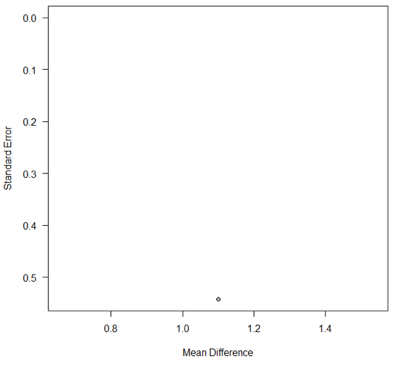


**7g HKA**

(a) MA vs KA, (b) MA vs rKA

(a) (b)


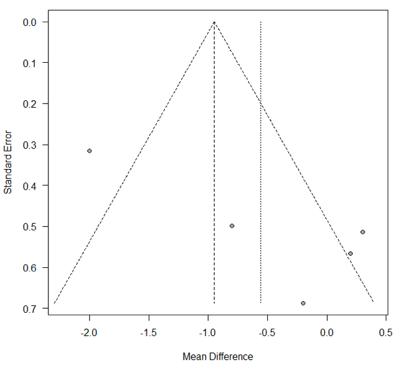

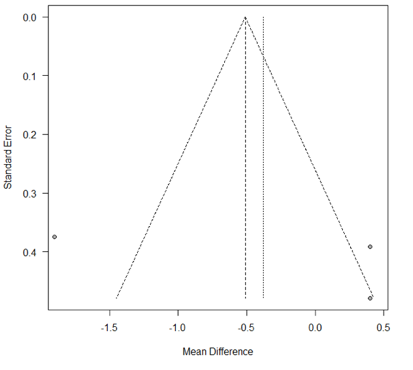

Supplement: Supplementary file 7 — Additional file 7. Funnel plots. [file 12891_2023_6448_MOESM7_ESM.docx]
