# Supplementary material for: Postoperative clinical outcomes for kinematically, restricted kinematically, or mechanically aligned total knee arthroplasty: a systematic review and network meta-analysis of randomized controlled trials
Source: BMC Musculoskelet Disord. 2023 Apr 24;24:322. doi: 10.1186/s12891-023-06448-0 (PMC10124064; doi:10.1186/s12891-023-06448-0)

**Additional file 8. Forest plot for PROMs in additional analysis**

PROMs, patient-reported outcome measures; CR, cruciate retaining; MA, mechanically aligned; KA, kinematically aligned; rKA, restricted kinematically aligned; SMD, standardised mean difference; CI, confidence intervals; MPP, medial parapatellar

**8a PROMs in CR insert studies.**


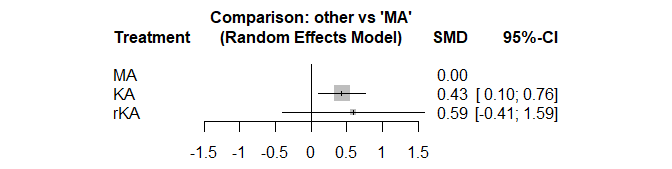


**8b PROMs in MPP approach studies.**


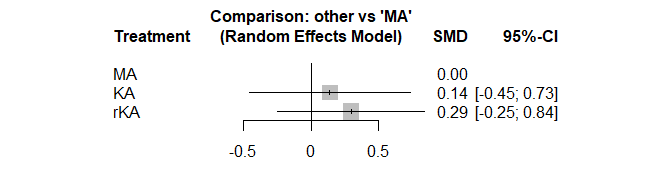


8c PROMs restricted to more than one-year follow-up studies.


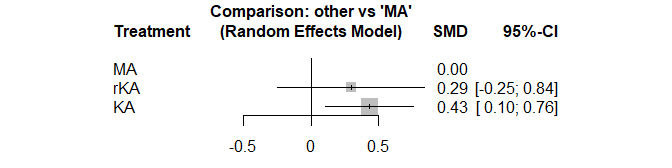

Supplement: Supplementary file 8 — Additional file 8. Forest plot for PROMs in additional analysis. [file 12891_2023_6448_MOESM8_ESM.docx]
